# Supplementary material for: Transformation-induced stress at telomeres is counteracted through changes in the telomeric proteome including SAMHD1
Source: Life Sci Alliance. 2018 Jul 17;1(4):e201800121. doi: 10.26508/lsa.201800121 (PMC6238619; doi:10.26508/lsa.201800121)
Supplement: Supplementary file 2 [file LSA-2018-00121_TableS2.pdf]

Table S2 Detailed analysis of the metaphase spreads shown in Fig 5C. Displayed are percentages of chromosome ends (also known as telomeres) with normal and abnormal telomeric FISH signals and a number of scored telomeres and metaphases per condition. Fragile = smeary or multiple telomeric signals; Outside = telomeric signal positioned outside the DAPI-stained chromatid end; Apposition = sister telomere association/fusion; Fusion = non-sister telomere association/fusion.

HLF-TSR	% of telomeres						No. of scored	
siRNA-replicate	Normal	Fragile	Loss	Outside	Apposition	Fusion	Telomeres	Metaphases
siGFP-1	94.1	4.7	0.5	0.1	0.7	0.0	2082	19
siGFP-2	95.1	3.0	0.7	0.1	1.2	0.0	1828	18
siGFP-3	92.8	6.2	0.2	0.0	0.7	0.0	2157	15
siSAMHD1-1	92.0	6.7	1.0	0.2	0.1	0.0	3730	28
siSAMHD1-2	94.2	4.8	0.8	0.2	0.0	0.1	1951	24
siDCLRE1B-1	92.0	5.7	0.7	0.0	1.6	0.0	1740	28
siDCLRE1B-2	90.1	8.4	0.9	0.1	0.5	0.0	3935	24
siTMPO-1	94.6	4.6	0.6	0.0	0.1	0.1	3508	28
siTMPO-2	95.1	4.0	0.7	0.2	0.1	0.0	2522	24
siNPAT-1	95.5	3.9	0.5	0.1	0.0	0.0	2163	28
siNPAT-2	93.9	5.0	1.0	0.2	0.0	0.0	505	24
siPARP9-1	93.1	6.1	0.5	0.1	0.0	0.2	3058	28
siPARP9-2	92.2	6.6	0.7	0.1	0.4	0.1	1701	24
siPNUTS-1	87.8	11.3	0.6	0.2	0.0	0.0	3128	28
siPNUTS-2	89.3	10.0	0.4	0.1	0.2	0.0	3354	24
siSMCHD1-1	89.1	10.3	0.6	0.0	0.0	0.0	3230	28
siSMCHD1-2	93.1	6.3	0.3	0.0	0.2	0.0	2469	24

HeLa	% of telomeres						No. of scored	
siRNA	Normal	Fragile	Loss	Outside	Apposition	Fusion	Telomeres	Metaphases
siGFP	93.2	6.3	0.0	0.1	0.5	0.0	1739	32
siSAMHD1	91.5	7.5	0.2	0.6	0.3	0.0	2961	32
siDCLRE1B	92.3	6.4	0.4	0.6	0.3	0.0	3801	32
siTMPO	88.7	10.5	0.4	0.3	0.1	0.0	3852	32
siNPAT	81.9	17.5	0.4	0.1	0.1	0.0	2091	32
siPARP9	89.2	10.3	0.2	0.3	0.0	0.0	2692	32
siPNUTS	83.7	16.2	0.1	0.1	0.0	0.0	1212	26
siSMCHD1	89.5	8.0	0.2	2.0	0.3	0.0	1874	32
