# Supplementary material for: Transformation-induced stress at telomeres is counteracted through changes in the telomeric proteome including SAMHD1
Source: Life Sci Alliance. 2018 Jul 17;1(4):e201800121. doi: 10.26508/lsa.201800121 (PMC6238619; doi:10.26508/lsa.201800121)
Supplement: Supplementary file 3 [file LSA-2018-00121_TableS3.pdf]

Table S3 Detailed analysis of the metaphase spreads shown in Fig 6. Displayed are percentages of chromosome ends (also known as telomeres) with normal and abnormal telomeric FISH signals and a number of scored telomeres and metaphases per condition. Fragile = smeary or multiple telomeric signals; Outside = telomeric signal positioned outside the DAPI-stained chromatid end; Apposition = sister telomere association/fusion; Fusion = non-sister telomere association/fusion.

			% of telomeres							
	shSAMHD1	shTRF1 or Aphidicolin	Normal	Fragile	Loss	Outside	Apposition	Fusion	Telomeres	Metaphases
Replicate 1	EV	EV	96.3	2.7	0.2	0.2	0.6	0.0	4062	27
	#2	EV	88.9	9.6	0.3	0.7	0.4	0.0	4702	27
	#4	EV	89.1	9.1	0.3	0.7	0.7	0.0	4912	27
	EV	shTRF1	88.1	7.6	0.5	2.1	1.1	0.6	4455	27
	#2	shTRF1	83.5	6.2	0.8	8.1	1.1	0.1	5423	27
	#4	shTRF1	87.6	6.5	0.3	4.6	0.9	0.0	5327	27
Replicate 2	EV	EV	89.1	10.2	0.2	0.0	0.4	0.1	5258	27
	#2	EV	85.6	14.1	0.1	0.1	0.1	0.0	6278	27
	#4	EV	84.8	13.1	0.6	0.4	1.2	0.0	2710	27
	EV	shTRF1	78.7	17.1	0.4	2.1	1.2	0.6	5605	27
	#2	shTRF1	79.8	11.9	0.2	7.0	1.0	0.1	5816	27
	#4	shTRF1	81.6	12.4	0.2	4.3	1.2	0.3	4009	27
Replicate 3	EV	EV	93.4	6.2	0.2	0.1	0.0	0.1	5319	27
	#2	EV	92.6	7.0	0.2	0.2	0.0	0.0	5624	27
	#4	EV	93.2	6.1	0.2	0.4	0.0	0.0	5624	27
	EV	shTRF1	89.9	6.4	0.4	0.9	1.4	0.9	5381	27
	#2	shTRF1	88.7	5.5	0.9	4.5	0.3	0.1	5345	27
	#4	shTRF1	89.1	6.0	0.8	2.9	0.4	0.7	5385	27
Single replicate	EV	-	93.2	6.5	0.2	0.1	0.0	0.0	6056	35
	#2	-	89.7	9.7	0.4	0.2	0.0	0.0	3401	17
	#4	-	92.2	7.4	0.2	0.2	0.0	0.0	3794	21
	EV	Aph.	89.5	9.8	0.4	0.0	0.3	0.0	3557	37
	#2	Aph.	89.4	9.7	0.3	0.1	0.4	0.0	3362	28
	#4	Aph.	94.6	4.5	0.2	0.2	0.4	0.1	3254	33
