# Supplementary material for: Transformation-induced stress at telomeres is counteracted through changes in the telomeric proteome including SAMHD1
Source: Life Sci Alliance. 2018 Jul 17;1(4):e201800121. doi: 10.26508/lsa.201800121 (PMC6238619; doi:10.26508/lsa.201800121)
Supplement: Supplementary file 4 [file LSA-2018-00121_TableS4.pdf]

Table S4 List of primers for RT–qPCR of TERRA.

Primer ID	Primer sequence	Target
MF604_tRT	CCCTAACCCTAACCCTAACCCTAACCCTAA	RT oligo for human TERRA, as telomeric repeats.
MF629F	AGCCACATCGCTCAGACAC	GAPDH
MF630R	GCCCAATACGACCAAATCC	GAPDH
PN379	TAAGCCGAAGCCTAACTCGTGTC	Amplification for 2p subtelomere.
PN378	GTAAAGGCGAAGCAGCATTCTCC	Amplification for 2p subtelomere.
MF654R	ACATGAGGAATGTGGGTGTTAT	Amplification for 9p subtelomere.
MF653F	GAGATTCTCCCAAGGCAAGG	Amplification for 9p subtelomere.
MF624R	GATCCCACTGTTTTTATTACTGTTCCT	Amplification for 17p subtelomere.
MF623F	GGGACAGAAGTGGATAAGCTGATC	Amplification for 17p subtelomere.
PN446R	TTTGTTCACTGTCGATGCG	Amplification for 20q subtelomere.
PN445F	GCAGCTTTCTCAGCACAC	Amplification for 20q subtelomere.
MF807F	CGGCCGAGTTGCGTTCTCG	Amplification for Xq subtelomere.
MF811R	GCACATGAGGAATGTGGGTG	Amplification for Xq subtelomere.
MF812F	ATTCTCCTCAGGTCAGACCCG	Amplification for 15 subtelomere.
MF814R	CTAACCGTGACCCTGACCCCG	Amplification for 15 subtelomere.
