# Supplementary material for: Transformation-induced stress at telomeres is counteracted through changes in the telomeric proteome including SAMHD1
Source: Life Sci Alliance. 2018 Jul 17;1(4):e201800121. doi: 10.26508/lsa.201800121 (PMC6238619; doi:10.26508/lsa.201800121)
Supplement: Supplementary file 5 [file LSA-2018-00121_TableS5.pdf]

Table S5. List of primers for RT–qPCR to verify candidate depletion.

Target	Primer ID	Target sequence	Source
DCLRE1B	JM013F	TGGAACCATCCTCTACACAGGT	This study*
	JM014R	GGATCTGTTTCCCCAGTGTCA	
GAPDH	JM051F	AGCCACATCGCTCAGACAC	(Feretzaki & Lingner, 2017)
	JM052R	GCCCAATACGACCAAATCC	
NPAT	JM077F	TCCCAGTATCTGTGGTTGGAC	This study*
	JM078R	CAGGTGCTGTCAAAGGCATA	
PARP9	JM065F	GGCAAAGAGGTCCAAGATGCTG	(Bachmann et al, 2014)
	JM066R	GCCTCACACATCTCTTCCACGT	
PNUTS	JM069F	AAAACGAGCACAGAACCAA	(Kavela et al, 2013)
	JM070R	GGCTCCAAAGAGGCTGTAT	
SAMHD1	JM019F	GAGTCTCGTTTTGAAAATCTTGGA	This study*
	JM020R	CCAATCGCTGGATATAACTAAGCA	
SMCHD1	JM057F	TAACAACTGGGCCGTGTATAGG	(Tang et al, 2014)
	JM058R	TGGTACTGGACGAACATATCCTG	
TMPO	JM039F	CGGAGTGAATCCTGGTCCTATT	This study*
	JM040R	CCATTCTGCCTTGTATTTTCTGC	

*Primers were designed using ProbeFinder for the Human Universal ProbeLibrary (www.universalprobelibrary.com) from Roche. Primers were designed to recognize common regions of genes that comprise multiple transcript variants.
